# Design of Differential Loudspeaker Line Array for Steerable Frequency-Invariant Beamforming

**DOI:** 10.3390/s24196277

**Published:** 2024-09-27

**Authors:** Yankai Zhang, Qian Xiang, Qiaoxi Zhu

**Affiliations:** 1 Anhui Digital Intelligent Engineering Research Center for Agricultural Products Quality Safety, Fuyang Normal University, Fuyang 236037, China; 2 Anhui Province Photovoltaic Industry Common Technology Research Center, Fuyang Normal University, Fuyang 236037, China; xiangqian@fynu.edu.cn; 3 Faculty of Engineering and IT, University of Technology Sydney, Sydney, NSW 2007, Australia; qiaoxi.zhu@gmail.com

**Keywords:** differential loudspeaker arrays, beam steering, frequency-invariant beamforming

## Abstract

Differential beamforming has attracted much research since it can utilize an array with a small aperture size to form frequency-invariant beampatterns and achieve high directional gains. It has recently been applied to the loudspeaker line array to produce a broadside frequency-invariant radiation pattern. However, designing steerable frequency-invariant beampatterns for the loudspeaker line array has yet to be explored. This paper proposes a method to design a steerable differential beamformer with a loudspeaker line array. We first determine the target differential beampatterns according to the desired direction, the main lobe width, and the beampattern order. Then, we transform the target beampattern into the modal domain for representation. The Jacobi-Anger expansion is subsequently used to design the beamformer so that the resulting beampattern matches the target differential beampattern. Furthermore, based on the criterion of minimizing the mean square error between the synthesized beampattern and the ideal one, a multi-constraint optimization problem, which compromises between the robustness and the mean square error, is formulated to calculate the optimal desired weighting vector. Simulations and experimental results show that the proposed method can achieve steerable frequency-invariant beamforming from 300 Hz–4 kHz.

## 1. Introduction

Beamforming is a fundamental technique in array signal processing and has garnered significant attention in the research [1,2,3,4,5,6]. In microphone arrays, beamforming suppresses spatial interferences and improves the output signal-to-noise ratio [7,8,9,10,11,12]. In loudspeaker arrays, beamforming creates highly directional beampatterns, reducing room reflections and enhancing the desired sound’s delivery [13,14,15,16,17,18]. Array systems can be classified into two categories: additive arrays and differential arrays. Additive arrays generate directional beampatterns through the synchronize-and-add principle [19]. However, they cannot achieve high directivity at low frequencies with limited array apertures. In contrast, differential arrays, which respond to the spatial derivatives of the sound field, can generate narrow beampatterns and achieve high directional gains even with small apertures [20].

Differential arrays offer the advantages of compact size, frequency-invariant beampatterns, and high spatial directivity, and have been extensively studied in microphone array applications over the last two decades. Differential microphone arrays can be implemented in various geometric shapes, including line-shaped [20,21,22,23,24,25], planar [26,27,28,29,30,31,32,33], and volumetric designs [34,35,36,37]. Among these, the line-shaped differential microphone array (LDMA) is particularly well-studied due to its ease of integration with electronic devices. While most research focuses on directing the main lobe in the end-fire direction, this is often unsuitable for applications like smart TVs or tablets, where beam steering is needed, e.g., to accurately pick up voice commands from users speaking from different positions. Planar arrays, such as circular arrays [26,31,32] or concentric circular arrays [27,30,33], have much better steering capabilities. Based on the use of circular harmonics decomposition [32] and spherical harmonics decomposition [33], the beamformers can be designed to steer the frequency-invariant beampatterns in two-dimensional and three-dimensional spaces. Volumetric arrays, such as cube arrays [34,35,36] and spherical arrays [37], can also generate steerable differential beampatterns in a three-dimensional space. Although planar arrays and volumetric arrays can solve the beam steering issue, their large size limits their application in certain scenarios. As a result, there is a great need to study the problem of beam steering with LDMAs.

Efforts have been made to design first- and second-order steerable LDMAs [38,39], including the development of general conditions for steerable differential beamformers [40]. However, the ideal beampattern can become ineffective due to improper null position selection. To address this, extremum and interference suppression constraints have been used in designing steerable LDMAs [41]. Nonetheless, selecting the optimal trade-off parameter between the directivity factor (DF) and interference suppression energy remains challenging. Recently, a novel method was proposed using both omnidirectional and directional microphones to design fully steerable LDMAs, achieving steering- and frequency-invariant beampatterns with a linear super array (LSA) [42,43]. However, mismatches between omnidirectional and directional microphones can impact beamformer performance in practical applications.

Differential beamformers have also been applied to linear loudspeaker arrays to produce highly directional patterns [44,45,46,47,48,49,50,51]. For example, a three-element line array with a second-order differential broadside beam has been studied to create a near-field sound zone in car cabins [46]. For higher-order broadside differential beampatterns, a null-constrained method has been proposed as a convenient approach for designing differential beamformers [47]. However, its frequency-invariant beampattern is limited, which may be undesirable for certain applications. Our recent research extends the null-constrained method using a series expansion approach for broadside linear loudspeaker arrays, enhancing the beamformer’s robustness and better preserving the frequency-invariant beampattern [50,51]. In a study of beam steering for differential loudspeaker arrays (DLAs), a linear loudspeaker array composed of miniature omnidirectional speakers and dipole speakers is used to design steerable first-order DLAs [49]. Our recent study develops steerable frequency-invariant differential beampatterns using a circular loudspeaker array with a rigid baffle [48]. However, miniature loudspeakers and circular arrays are not widely applicable, such as in public broadcasting and outdoor sound reinforcement scenarios. Therefore, there is a need to explore beam steering with loudspeaker line arrays.

In this paper, we propose a method for designing steerable differential beamformers using a loudspeaker line array. The key contributions include (1) calculating the target steerable differential beampattern based on the desired direction, main lobe width, and beampattern order; (2) formulating the desired differential beampattern in the modal domain for representation; and (3) developing a multi-constraint optimization problem, which compromises the white noise gain (WNG) and the mean square error (MSE) between the synthesized and ideal beampatterns, to design a steerable frequency-invariant beamformer using the Jacobi-Anger expansion. Both simulation and experimental studies are provided. Although this work focuses on differential loudspeaker line arrays, the proposed method is also applicable to differential microphone arrays, making it suitable for designing LDMAs with steerable differential beampatterns for high-quality acoustic signal acquisition.

Notice that some methods were developed in the literature to achieve a compromise between WNG and DF of the differential beamformers with LDMAs. In [52], a simple theory and a novel differential beamforming method are proposed to compromise the white noise gain (WNG), the directivity factor (DF), and the front-to-back ratio (FBR) of the beamformer. In [53], the authors present a study on theory and methods based on the null constrained fixed beamformer to achieve the optimal and fundamental compromise between WNG and DF, e.g., to find a beamformer that maximizes the DF and whose WNG is equal to a preset value. But these beamforming methods with LDMAs only consider the desired signal that comes from the end-fire direction, and the designed beamformer does not have the capability for beam steering. The focus of this work is on how to design differential beampatterns with steering capability using a linear array and, once the desired beampattern is determined, how to achieve a compromise between WNG and MSE in the modal domain. Therefore, the method proposed in this paper is significantly different from the methods presented in the aforementioned literature.

The paper is organized as follows: Section 2 introduces the signal model, problem formulation, and key definitions. Section 3 details the proposed method for designing steerable frequency-invariant beampatterns with a linear array. Section 4 presents simulations with design examples and discusses the impacts of constraint parameters and element mismatch, as well as a performance comparison with existing methods. Section 5 provides experimental results that align with the simulations, validating the proposed method. Finally, Section 6 concludes the paper.

## 2. Problem Formulation

**Figure 1:**
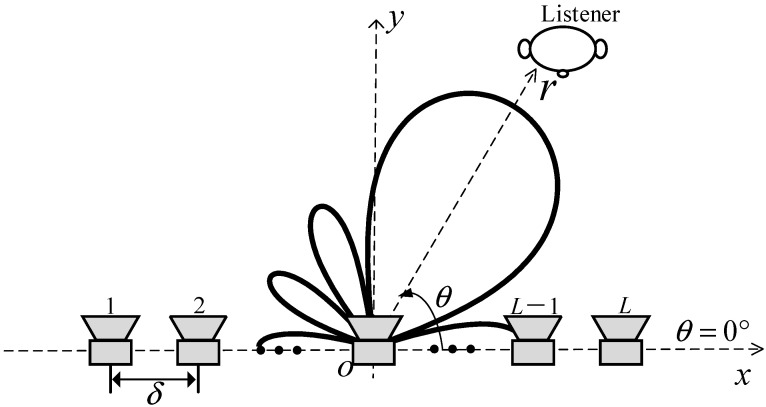
The loudspeaker line array with a steerable differential radiation pattern.

Figure 1 illustrates a loudspeaker array designed to radiate a steerable differential beampattern towards a listener. The line array, consisting of *L* loudspeakers with an interelement spacing δ, lies on the *x* axis. The loudspeaker array is centered at the origin of the coordinate system. Each loudspeaker is modeled as an omnidirectional point source. The far-field sound pressure at a listener position (r,θ) generated by the loudspeaker array can be expressed as

(1) $$p(k,r,\theta )\approx \frac{e^{ikr}}{4\pi r}\sum_{l=1}^{L}w_{l}^{*}(k)e^{-ikx_{l}\cos\theta },$$

where i is the imaginary unit, with $i^{2}=-1$, k=2πf/c is the wave number, *f* denotes the frequency, *c* is the speed of sound in air, the superscript ${\left(\cdot \right)}^{*}$ is the complex-conjugate operator, $w_{l}(k)$ denotes the weight of the *l*-th loudspeaker at $\left(x_{l},0\right)$, where $x_{l}=-(L+1)\delta /2+l\delta$, l is the loudspeaker index, *l* = 1, 2, …, *L*, *r* is the distance from the origin of the coordinate system to the listener, and the angle θ is defined with the respect to the positive *x* axis.

In the far-field, the directivity of the loudspeaker array does not change with the distance *r*. To evaluate the directivity of radiation, the normalized far-field radiation pattern is as follows:

(2) $$B(k,\theta )=p(k,r,\theta )/(e^{ikr}/4\pi r)=\sum_{l=1}^{L}w_{l}^{*}(k)e^{-ikx_{l}\cos\theta }$$

This can be written in a vector form,

(3) $$B(k,\theta )=w^{H}(k)g(k,\theta ),$$

where

(4) $$w(k)={\left[w_{1}(k),\ldots ,w_{L}(k)\right]}^{T},$$

(5) $$g(k,\theta )={\left[e^{-ikx_{1}\cos\theta },\cdots ,e^{-ikx_{L}\cos\theta }\right]}^{T},$$

the superscripts ${\left(\cdot \right)}^{H}$ and ${\left(\cdot \right)}^{T}$ are the conjugate-transpose operator and the transpose operator, respectively, and w is the beamforming filter to be designed to achieve a steerable differential radiation pattern.

Apart from the normalized far-field radiation pattern, there are two commonly used metrics to evaluate the performance of a beamformer: white noise gain (WNG) and directivity factor (DF). The WNG quantifies the robustness of the beamformer against the white noise,

(6) $$\mathrm{WNG}(k)=\frac{{\left|B(k,\theta_{s})\right|}^{2}}{w^{H}(k)w(k)},$$

where $\theta_{s}$ is the desired radiation direction. For the loudspeaker array, the WNG also represents the radiation efficiency of the array.

The DF evaluates the directional characteristics of the beamformer. The two-dimensional DF is defined as the ratio of the sound power radiated in the desired direction to the average sound power across the half-plane in front of the loudspeaker array, and is expressed as

(7) $$\mathrm{DF}(k)=\frac{\pi {\left|B(k,\theta_{s})\right|}^{2}}{\int_{0}^{\pi }{\left|B(k,\theta )\right|}^{2}d\theta }=\frac{{\left|B(k,\theta_{s})\right|}^{2}}{w^{H}(k)\Gamma (k)w(k)},$$

where

(8) $$\Gamma (k)=\frac{1}{\pi }\int_{0}^{\pi }g(k,\theta )g^{H}(k,\theta )d\theta (1)$$

is an L×L square matrix, whose elements are ${\left[\Gamma \right]}_{m,n}(k)=\begin{array}{cc} J_{0}[k(i-j)\delta ], & m,n\in \{1,2,\ldots ,L\} \end{array}$. Here, $J_{0}(\cdot )$ is the zero-order Bessel function of the first kind. For brevity, we will omit the dependence on *k* in the following text.

## 3. Methods

The objective of the proposed method is to form a directional steerable loudspeaker array, such that the energy radiated by the array is mainly concentrated in the main lobe area, while minimizing the output energy of the sidelobe area. It is achieved in three steps: (1) calculating the target radiation pattern according to the radiation steering angle, main lobe area and sidelobe area; (2) formulating the target radiation pattern in the modal domain; and (3) designing the steerable differential beamformer using the modal matching with the Jacobi-Anger expansion.

### 3.1. Target Radiation Pattern with the Main Lobe Steering

The ideal *N*-th order differential beampattern for a differential microphone array is

(9) $${\tilde{B}}^{\left(N\right)}(\theta )=\sum_{n=0}^{N}\alpha_{N,n}\cos^{n}\theta ,$$

which assumes the microphone spacing is much smaller than the wavelength, and $\alpha_{N,n},n=0,1,\ldots ,N$ are real coefficients. To allow main lobe steering to the desired direction $\theta_{s}$, two conditions should be satisfied: that ${\tilde{B}}^{\left(N\right)}(\theta_{s})=1$ and $\partial {\tilde{B}}^{\left(N\right)}(\theta_{s})/\partial \theta_{s}=0$. In the vector form,

(10) $$A^{T}\alpha_{N}=b,$$

where

(11) $$A={\left[\begin{array}{cccc} 1 & \cos\theta_{s} & \ldots & \cos^{N}\theta_{s}\\ 0 & \sin\theta_{s} & \ldots & N\cos^{N-1}\theta_{s}\sin\theta_{s} \end{array}\right]}^{T},$$

(12) $$\alpha_{N}={\left[\begin{array}{cccc} \alpha_{N,0} & \alpha_{N,1} & \ldots & \alpha_{N,N} \end{array}\right]}^{T},b={\left[\begin{array}{cc} 1 & 0 \end{array}\right]}^{T}.$$

Also, (9) in the vector form is

(13) $${\tilde{B}}^{\left(N\right)}(\theta )=\alpha_{N}^{T}c_{N},$$

where

(14) $$c_{N}={\left[\begin{array}{cccc} 1 & \cos\theta & \ldots & \cos^{N}\theta \end{array}\right]}^{T}.(2).$$

The corresponding beam power can be expressed as

(15) $${\left|{\tilde{B}}^{\left(N\right)}(\theta )\right|}^{2}=\alpha_{N}^{T}c_{N}c_{N}^{T}\alpha_{N}=\alpha_{N}^{T}C\alpha_{N},$$

where

(16) $$C=c_{N}c_{N}^{T}.$$

Due to the acoustic reciprocity principle, our proposed method applies Equation (13) as the ideal *N*-th order differential radiation pattern for a differential loudspeaker array. It enables precise steering of sound propagation towards the desired direction $\theta_{s}$. Additionally, it is necessary to minimize the radiation of sound energy in the sidelobe area, defined as

(17) $$\Theta_{SL}\in \left[\begin{array}{cc} 0^{\circ }, & \theta_{s}-\Delta \end{array}/2\right]\cup \left[\begin{array}{cc} \theta_{s}+\Delta /2, & 180^{\circ } \end{array}\right],$$

where Δ is the main lobe width. Therefore, once the desired direction and main lobe width are determined, the optimal solution $\alpha_{N}$ can be obtained by solving the following optimization problem:

(18) $$\begin{array}{cc} \underset{\alpha_{N}}{\min}{\left|{\tilde{B}}^{\left(N\right)}(\Theta_{SL})\right|}^{2} & s.t. \end{array}\begin{array}{cc} & A^{T}\alpha_{N}=b \end{array}.$$

This can be solved using the CVX toolbox [54]. Moreover, the selection of the main lobe width Δ and the desired direction $\theta_{s}$ must satisfy

(19) $$\Delta \leq 2\cdot \min(\theta_{s},180^{\circ }-\theta_{s}).$$

**Figure 2:**
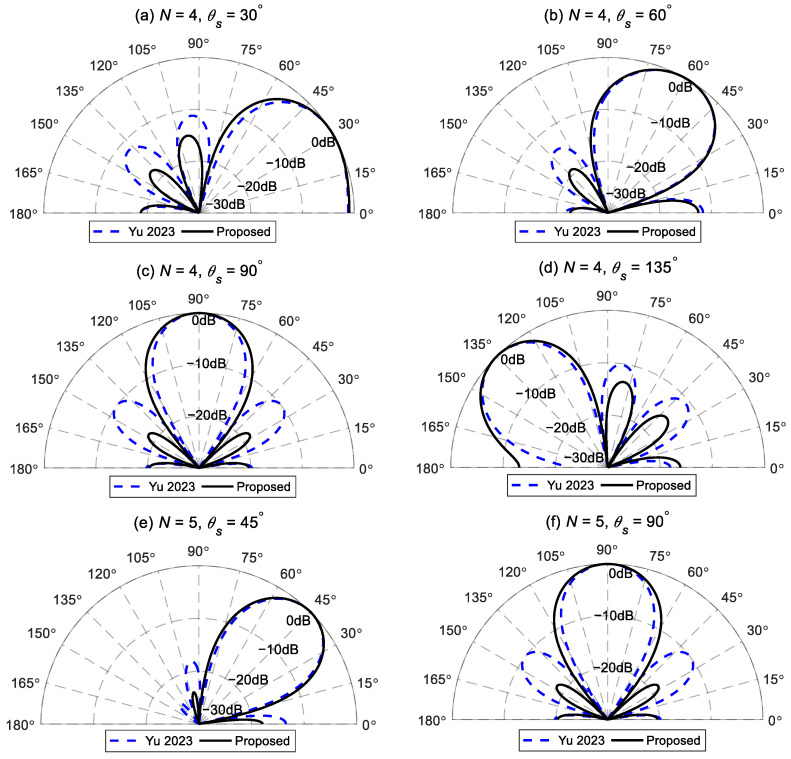
Comparison of the *N*-th order target radiation patterns designed by the method stated in Ref. [41] and the proposed method at different desired angels: (**a**) N=4, $\theta_{s}=30^{\circ }$, (**b**) N=4, $\theta_{s}=60^{\circ }$, (**c**) N=4, $\theta_{s}=90^{\circ }$, (**d**) N=4, $\theta_{s}=135^{\circ }$, (**e**) N=5, $\theta_{s}=45^{\circ }$, and (**f**) N=5, $\theta_{s}=90^{\circ }$.

The optimal target radiation pattern can be obtained by substituting the resultant into (13). Figure 2 provides examples of target radiation patterns designed using the above method at different desired angles. This indicates that the proposed method can design effective radiation patterns for different orders in various desired directions. Figure 2 also presents the target radiation patterns of the same order and desired directions designed using the method stated in Ref. [41]. Yu’s method finds the optimal vector $\alpha_{N}$ by maximizing the directivity factor of the beampattern, under the distortionless constraint and the extremum constraint in the desired direction (10). This method needs a regularization parameter to trade off the radiation power in the end-fire direction and the spatial average of the radiated power over the entire space. The regularization parameter is set to 0.15, as given in Ref. [41] and applied in the following simulation.

Figure 2a–d present the comparison results of the fourth-order target differential radiation patterns at desired angles of 30°, 60°, 90°, and 135°, using Yu’s method and the proposed method, respectively. The main lobe width Δ is set to 60°. Though both methods can design effective fourth-order differential radiation patterns at the desired angles, the proposed method has smaller sidelobes (excluding those in the end-fire direction). This is because Yu’s method adjusts the regularization parameter to balance the radiated energy in the end-fire direction and the average over the entire space, when designing the target radiation pattern. Since this method does not account for the energy distribution of sidelobes other than the end-fire direction, it can result in excessively large sidelobes. Thus, the regularization parameter selection is required for different desired directions. However, selecting the appropriate regularization parameter is quite challenging, which hinders the practical application of this method. In contrast, the proposed method designs the target radiation patterns by minimizing the total energy in the sidelobe area, which includes the end-fire direction. As a result, the target beampattern radiates less energy into the sidelobe area. Additionally, once the desired direction and main lobe width are determined, we can design the optimal radiation pattern without adjusting other parameters, facilitating practical applications.

Figure 2e,f further compare the fifth-order target radiation patterns by Yu’s and the proposed methods, with the desired directions of $\theta_{s}=45^{\circ }$ and $\theta_{s}=90^{\circ }$, respectively. The above findings hold. In addition, when the desired direction is in the broadside direction, $\theta_{s}=90^{\circ }$, the fifth-order target radiation pattern (Figure 2f) is the same as the fourth-order target pattern (Figure 2c). This occurs because the target radiation pattern in the broadside direction is symmetrical along the y axis. As a result, the odd terms of the optimal vector $\alpha_{N}$ are zero, leading to pairs of nulls in the target beampattern that are symmetrically distributed along the *y* axis. Therefore, when designing *N*-th order broadside radiation patterns (where *N* is odd), the process automatically reduces to designing an (*N* − 1)-th order pattern.

### 3.2. Formulating the Target Radiation Pattern in the Modal Domain

Using the *N*-th order target radiation pattern obtained in Section 3.1, we will transform the ideal radiation pattern from Equation (9) into the modal domain, depending on the parity of *N*.

#### 3.2.1. *N* Is Even

Let $N=2\tilde{N}$ and $\alpha_{N,-1}=0$, (9) will be

(20) $${\tilde{B}}^{\left(N\right)}(\theta )=\sum_{n=0}^{N}\alpha_{N,n}\cos^{n}\theta =\sum_{n=0}^{\tilde{N}}\alpha_{N,2n}\cos^{2n}\theta +\sum_{n=0}^{\tilde{N}}\alpha_{N,2n-1}\cos^{2n-1}\theta .$$

According to the following relations [55],

(21) $$\cos^{2n}\theta =\frac{1}{2^{2n}}\left\{\sum_{t=0}^{n-1}2\left(\begin{array}{c} 2n\\ t \end{array}\right)\cos2\left(n-t\right)\theta +\right.\left.\left(\begin{array}{c} 2n\\ n \end{array}\right)\right\},$$

(22) $$\cos^{2n-1}\theta =\frac{1}{2^{2n-2}}\left\{\sum_{t=0}^{n-1}\left(\begin{array}{c} 2n-1\\ t \end{array}\right)\cos\left[2\left(n-t\right)-1\right]\theta \right\},$$

where $\left(\begin{array}{c} \cdot \\ \cdot \end{array}\right)$ is combinations. Combining the Euler’s formula, i.e.,

(23) $$\cos(n\theta )=\frac{e^{in\theta }+e^{-in\theta }}{2},$$

then (20) can be expressed as

(24) $${\tilde{B}}^{\left(N\right)}(\theta )=\sum_{n=-N}^{N}\gamma_{n}e^{in\theta },$$

where

(25) $$\gamma_{n}=\left\{\begin{array}{c} \begin{array}{cc} \sum_{p=\frac{\left|n\right|+1}{2}}^{\tilde{N}}\alpha_{N,2p-1}{\tilde{\eta }}_{\frac{n+\mathrm{sgn}(n)}{2}}(p) & n=\pm 1,\pm 3,\ldots ,\pm (2\tilde{N}-1)\begin{array}{cc} & , \end{array} \end{array}\\ \begin{array}{cc} \sum_{p=\left|n\right|/2}^{\tilde{N}}\alpha_{N,2p}\eta_{n/2}(p) & n=0,\pm 2,\pm 4 \end{array},\ldots ,\pm 2\tilde{N}\begin{array}{cc} & , \end{array} \end{array}\right.$$

(26) $${\tilde{\eta }}_{t}(n)=\left\{\begin{array}{c} \begin{array}{cc} 0 & t=0\begin{array}{cc} & , \end{array} \end{array}\\ \begin{array}{cc} \frac{1}{2^{2n-1}}\left(\begin{array}{c} 2n-1\\ n-\left|t\right| \end{array}\right) & t=\pm 1,\pm 2,\ldots ,\pm n\begin{array}{cc} & , \end{array} \end{array} \end{array}\right.$$

(27) $$\mathrm{sgn}(t)=\left\{\begin{array}{c} \begin{array}{cc} 1 & t>0\begin{array}{cc} & , \end{array} \end{array}\\ \begin{array}{cc} 0 & t=0\begin{array}{cc} & , \end{array} \end{array}\\ \begin{array}{cc} -1 & t<0\begin{array}{cc} & , \end{array} \end{array} \end{array}\right.$$

(28) $$\eta_{t}(n)=\frac{1}{2^{2n}}\left(\begin{array}{c} 2n\\ n-\left|t\right| \end{array}\right)\begin{array}{cc} & t=0,\pm 1,\ldots ,\pm n. \end{array}$$

Here, $\left|\cdot \right|$ represents absolute value.

#### 3.2.2. *N* Is Odd

Let N’=(N+1)/2 and

(29) $$\alpha_{2N’,n}=\left\{\begin{array}{c} \begin{array}{cc} \alpha_{N,n} & n=0,1,\ldots ,N\begin{array}{cc} & , \end{array} \end{array}\\ 0\begin{array}{cc} & n=N+1\begin{array}{cc} & , \end{array} \end{array} \end{array}\right.$$

and then we can express the odd (*N*-th) order target radiation pattern as a form of the even (2N’) order target beampattern:

(30) $${\tilde{B}}^{\left(N\right)}(\theta )={\tilde{B}}^{\left(2N’\right)}(\theta )=\sum_{n=0}^{2N’}\alpha_{2N’,n}\cos^{n}\theta =\sum_{n=0}^{N’}\alpha_{2N’,2n}\cos^{2n}\theta +\sum_{n=0}^{N’}\alpha_{2N’,2n-1}\cos^{2n-1}\theta .$$

Similar to the derivation steps in Section 3.2.1, we can formulate (30) in the modal domain with a symmetric form,

(31) $${\tilde{B}}^{\left(N\right)}(\theta )={\tilde{B}}^{\left(2N’\right)}(\theta )=\sum_{n=-2N’}^{2N’}\gamma_{n}e^{in\theta },$$

where

(32) $$\gamma_{n}=\left\{\begin{array}{c} \begin{array}{cc} \sum_{p=\frac{\left|n\right|+1}{2}}^{N’}\alpha_{2N’,2p-1}{\tilde{\eta }}_{\frac{n+\mathrm{sgn}(n)}{2}}(p) & n=\pm 1,\pm 3,\ldots ,\pm (2N’-1) \end{array}\\ \begin{array}{cc} \sum_{p=\left|n\right|/2}^{N’}\alpha_{2N’,2p}\eta_{n/2}(p) & n=0,\pm 2,\pm 4 \end{array},\ldots ,\pm 2N’ \end{array}\right.,$$

Here, ${\tilde{\eta }}_{t}(n)$, sgn(t), and $\eta_{t}(n)$ are defined in (26), (27), and (28), respectively.

### 3.3. Beamformer Design

The objective is to determine the loudspeaker weighting so that the array’s radiation pattern closely approximates the target radiation pattern. Based on (2) and (24), we use the Jacobi-Anger series expansion method to accomplish this task.

#### 3.3.1. Modal Matching Method with Maximum WNG

In the series expansion method, the Jacobi–Anger expansion is used to decompose the resulting radiation pattern into a linear combination of circular harmonics. The Jacobi-Anger expansion is

(33) $$e^{-i\sigma \cos\theta }=\sum_{n=-\infty }^{+\infty }\beta_{n}(\sigma )e^{in\theta },$$

where $\beta_{n}(\sigma )={\left(-i\right)}^{n}J_{n}(\sigma )$ and $J_{n}(\cdot )$ is the N-th order Bessel function of the first kind. Substituting (33) into (2), we obtain

(34) $$B(\theta )=\sum_{l=1}^{L}w_{l}^{*}\sum_{n=-\infty }^{+\infty }\beta_{n}(kx_{l})e^{in\theta }.$$

In order to obtain an *N*-th order target radiation pattern, the infinite summation in (34) is truncated to the order *N*:

(35) $$B(\theta )\approx \sum_{n=-N}^{N}e^{in\theta }\sum_{l=1}^{L}\beta_{n}(kx_{l})w_{l}^{*}.$$

When the normalized far-field radiation pattern in (35) is inconsistent with the target radiation pattern in (24), the following is true:

(36) $$\sum_{l=1}^{L}\beta_{n}(kx_{l})w_{l}^{*}=\gamma_{n}\begin{array}{cc} & n=0,\pm 1,\ldots ,\pm N. \end{array}$$

For the design of a steerable differential beamformer, the distortionless constraint in the desired direction is needed:

(37) $$w^{H}g(\theta_{s})=1.$$

Combing (36) and (37) yields

(38) Φw=ν,

where

(39) $$\Phi ={\left[\begin{array}{ccc} \beta_{-N} & \ldots & \begin{array}{cc} \beta_{N} & g(\theta_{s}) \end{array} \end{array}\right]}^{H},$$

(40) $$\beta_{n}={\left[\begin{array}{ccc} \beta_{n}(kx_{1}) & \cdots & \beta_{n}(kx_{L}) \end{array}\right]}^{T},$$

and

(41) $$\nu ={\left[\begin{array}{cc} \begin{array}{ccc} \gamma_{-N} & \ldots & \begin{array}{cc} \gamma_{0} & \begin{array}{cc} \ldots & \gamma_{N} \end{array} \end{array} \end{array} & 1 \end{array}\right]}^{T}.$$

Using the symmetric property of the Bessel function, $i^{n}J_{n}(\cdot )=i^{-n}J_{-n}(\cdot )$, (38) can be simplified to

(42) $$\tilde{\Phi }w=\tilde{\nu },$$

where

(43) $$\tilde{\Phi }={\left[\begin{array}{ccc} \beta_{0} & \cdots & \begin{array}{cc} \beta_{N} & g(\theta_{s}) \end{array} \end{array}\right]}^{H}$$

is $\left(N+2\right)\times L$ full matrix and

(44) $$\tilde{\nu }={\left[\begin{array}{cc} \begin{array}{ccc} \gamma_{0} & \ldots & \gamma_{N} \end{array} & 1 \end{array}\right]}^{T}.$$

With $L>\left(N+2\right)$ loudspeakers to generate an *N*-th order steerable target radiation pattern, one approach to design the beamformer is to maximize WNG with the constraints of (42). Due to (37), (6) can be written as $\mathrm{WNG}=1/w^{H}w$.

The optimization problem can be formulated as

(45) $$\begin{array}{cc} \underset{w}{\min}w^{H}w & \begin{array}{cc} s.t. & \tilde{\Phi }w=\tilde{\nu } \end{array} \end{array}.$$

The solution is

(46) $$w={\tilde{\Phi }}^{H}{\left(\tilde{\Phi }{\tilde{\Phi }}^{H}\right)}^{-1}\tilde{\nu }.$$

#### 3.3.2. Modal Matching Method with WNG Constraint

Since the resulting beamformer (46) is obtained with some approximations, the mean square error (MSE) is adopted to evaluate the accuracy of these approximations. The MSE of the approximations of the beamformer to the *N*-th order target differential radiation pattern is defined as

(47) $$d(w)=\frac{1}{\pi }\int_{0}^{\pi }{\left|w^{H}g(\theta )-{\tilde{B}}^{\left(N\right)}(\theta )\right|}^{2}d\theta .$$

Substituting (9) into (47), the MSE can be written as a quadratic function,

(48) $$d(w)=w^{H}\Gamma w-w^{H}q-q^{H}w+\xi ,$$

where

(49) $$q=\frac{1}{\pi }\int_{0}^{\pi }g(\theta ){\tilde{B}}^{\left(N\right)}(\theta )d\theta =Q\alpha_{N},$$

(50) $$Q=\frac{1}{\pi }\int_{0}^{\pi }g(\theta )c_{N}^{T}d\theta ,$$

(51) $$\xi =\int_{0}^{\pi }{\left|{\tilde{B}}^{\left(N\right)}(\theta )\right|}^{2}d\theta =\alpha_{N}^{T}\tilde{C}\alpha_{N},$$

(52) $$\tilde{C}=\frac{1}{\pi }\int_{0}^{\pi }c_{N}c_{N}^{T}d\theta .$$

The matrix Γ is the array spatial correlation matrix, which is defined in (8). Q is a complex matrix. Its (*m*, *n* + 1)th (*m* = 1, 2, …, *L*; *n* = 0, 1, …, *N*) element is

(53) $$Q_{m,n+1}=\frac{1}{2^{n-1}}\sum_{p=0}^{\left\lfloor n/2\right\rfloor }\left(\begin{array}{c} n\\ p \end{array}\right)i^{n-2p}J_{n-2p}(-kx_{m})-\frac{1}{2^{n}}\delta_{0,n-2\left\lfloor n/2\right\rfloor }\left(\begin{array}{c} n\\ \left\lfloor n/2\right\rfloor \end{array}\right)i^{n-2\left\lfloor n/2\right\rfloor }J_{n-2\left\lfloor n/2\right\rfloor }(-kx_{m}),$$

where $\left\lfloor \cdot \right\rfloor$ represents the floor function applied to a real number, $J_{n}(\cdot )$ is the N-th order Bessel function of the first kind, and $\delta_{m,n}$ represents the Kronecker delta. $\tilde{C}$ is an (L+1)×(L+1) real matrix, whose (*m* + 1, *n* + 1)th element is

(54) $${\tilde{C}}_{m+1,n+1}=\frac{1}{\pi }\int_{0}^{\pi }\cos^{m+n}\theta d\theta =\frac{(1+{\left(-1\right)}^{\left(m+n\right)})\Gamma \left((1+m+n)/2\right)}{2\sqrt{\pi }\Gamma \left((2+m+n)/2\right)}\begin{array}{cc} & \left(m=0,1,\ldots ,N;n=0,1,\ldots ,N\right) \end{array},$$

where Γ(⋅) is the Euler’s Gamma function.

After calculating the optimal coefficients of the target differential beampattern (9) according to the actual requirements, the objective for designing the beamformer is twofold. On the one hand, we aim to design a resulting beampattern that approaches the target one as much as possible. On the other hand, we hope the system is robust enough to resist unknown channel mismatch in real applications. The MSE, defined in (47), measures how closely the synthesized beampattern approximates the target beampattern. The WNG, defined in (6), is a metric for measuring the robustness of the designed beamformer. Therefore, attention should be paid to these two metrics when designing the beamformer.

The solution in (46) gives the maximum WNG. The higher the value of the WNG, the more robust the radiation. However, it does not consider the MSE in the design of the beamformer. In practice, a WNG greater than a suitable value should be ensured. It indicates the beamformer is robust enough for usage. In addition, the degrees of freedom provided by the weighting functions can be used to minimize MSE. The optimization problem can be described as follows:

(55) $$\begin{array}{l} \begin{array}{cc} \underset{w}{\min} & w^{H}\Gamma w-w^{H}q-q^{H}w \end{array}\\ \begin{array}{cc} s.t. & \end{array}\tilde{\Phi }w=\tilde{\nu },\\ \begin{array}{ccc} & & w^{H}w\leq \varepsilon_{\mathrm{WNG}}. \end{array} \end{array}$$

The parameter $\varepsilon_{\mathrm{WNG}}$ represents the lower limit of the WNG of the designed beamformer. It is noteworthy that $\varepsilon_{\mathrm{WNG}}$ must not exceed $\varepsilon_{\mathrm{WNG}}^{\max}$, which is the WNG obtained from the solution in (46). The optimal weighting vector w can be obtained by solving the optimization problem in (55) using the CVX toolbox [54]. To clearly illustrate the design process of a steerable differential beampattern with a linear array, we provide a detailed design procedure in Algorithm 1.
**Algorithm 1** Steerable Frequency-Invariant Beamformer DesignStep 1:Calculate the ideal differential beampattern:Set $N,\theta_{s}$ and Δ according to the actual application scenarios.Calculate A and b based on (11) and (12); Discretize the sidelobe area $\Theta_{SL}$ based on (17) and calculate $C_{\Theta_{SL}}$ based on (16).Compute $\alpha_{N}$ using the following CVX code:cvx_begin   variable $\alpha_{N}$   minimize quad_form($\alpha_{N}$, $C_{\Theta_{SL}}$)   subject to      $A^{T}\alpha_{N}=b$cvx_endStep 2:Calculate the circular harmonic coefficients of the ideal differential beampattern and the upper limit of the WNG:Calculate $\gamma_{n}(n=0,\pm 1,\ldots \pm N)$ based on (25) or (32), according to the parity of N and $\alpha_{N}$ calculated in Step 1.Calculate $\tilde{\Phi }$ and $\tilde{\nu }$ based on (43) and (44).Compute the optimal solution with maximum WNG $w_{\mathrm{MWNG}}$ based on (46) and calculate the corresponding WNG $\varepsilon_{\mathrm{WNG}}^{\max}$ dB based on (6).Step 3:Obtain the optimal solution by solving the multi-constraint optimization problem:Calculate Γ, q and ξ based on (8), (49) and (51).Compute the optimal solution $w_{\mathrm{opt}}$ using the following CVX code:cvx_begin   variable $w_{\mathrm{opt}}$ complex   minimize (real ($w_{\mathrm{opt}}^{H}\Gamma w_{\mathrm{opt}}$) − real (2*$w_{\mathrm{opt}}^{H}q$) + ξ)   subject to      $\tilde{\Phi }w_{\mathrm{opt}}=\tilde{\nu }$      real ($w_{\mathrm{opt}}^{’}w_{\mathrm{opt}}$) <= 1/10^[(εWNGmax−2)/10]cvx_end

In this section, we present the proposed method for designing steerable differential beampatterns using a loudspeaker line array. Section 3.1 provides the method for calculating the steerable target differential beampatterns using convex optimization, which has not been discussed in the existing literature. In Section 3.2, the analytical expression for the desired steerable differential beampatterns in the modal domain is presented for the first time. Section 3.3.1 uses the Jacobi-Anger expansion to approximate the beampattern generated by the array to the desired one in the modal domain, which is similar to the method in [23,27,32]. Then, we propose a multi-constraint optimization based on the modal matching method in Section 3.3.2 to find a beamformer that minimizes the MSE while the WNG is no less than a preset value. Therefore, the proposed steerable differential loudspeaker line array method leverages the remaining degrees of freedom to minimize the error caused by the finite truncation of the Jacobi-Anger series, while maintaining the robustness of the beamformer. This is the key theoretical contribution of this paper.

## 4. Simulations

In this section, we will verify the effectiveness of the proposed method and investigate the performance of the beamformer through simulations. A linear array of 21 loudspeakers with a spacing of 0.04 m is used in the following simulation. The frequency range of interest is from 300 Hz to 4 kHz, which covers the frequency range of speech.

### 4.1. Performance Study and Comparison

We first conduct the simulations to verify the effectiveness of the proposed method. For ease of identification, we refer to the method proposed in Section 3.3.1 as the proposed method I and the method proposed in Section 3.3.2 as the proposed method II. The parameter $\varepsilon_{\mathrm{WNG}}$ used in the proposed method II is set to 0 dB in this simulation.

**Figure 3:**
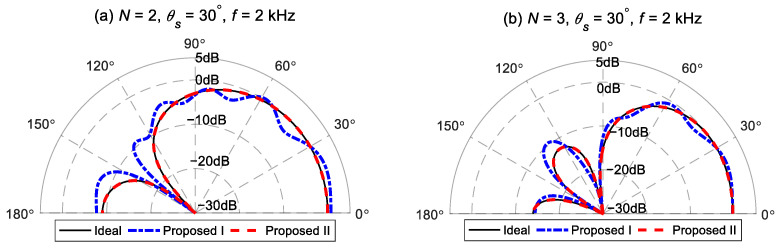
Comparisons between the different order beampatterns synthesized by the two proposed methods and the target beampattern at 2 kHz. (**a**) Second-order. (**b**) Third-order.

The second-order and third-order target radiation patterns, with the desired direction set as $\theta_{s}=30^{{}^{\circ}}$ and the main lobe width $\Delta =60^{{}^{\circ}}$, are synthesized by the proposed method I and the proposed method II, respectively. Figure 3 presents comparisons between the beampatterns synthesized using the two proposed methods and the target beampattern at 2 kHz. The target beampatterns have maximum values of one, which is located at the preset angle $\theta_{s}=30^{{}^{\circ}}$. The second-order target beampattern has a null at $138^{{}^{\circ}}$. The third-order target beampattern has two nulls at $100^{{}^{\circ}}$ and $154^{{}^{\circ}}$. Figure 3a shows the comparison result of the second-order beampattern. It is observed that the beampattern synthesized using the proposed method I deviates from the target beampattern. Despite the value of the synthesized beampattern being one at the preset direction, the values of the resulting beampattern near $20^{{}^{\circ}}$ and $60^{{}^{\circ}}$ are both greater than one. This indicates that although the distortionless constraint is satisfied in the proposed method I, the finite orders of modal matching cannot ensure the maximum value of the generated beampattern in the preset direction. However, the beampattern of the proposed method II matches well the target beampattern, shown with a solid black line. The same results can be seen in the comparison of the third-order beampattern shown in Figure 3b. It is noteworthy that, compared to the second-order synthesized beampattern, the third-order beampattern of the proposed method I is closer to the target beampattern. This is because the truncation error caused by (35) decreases as the order of the target beampattern increases.

**Figure 4:**
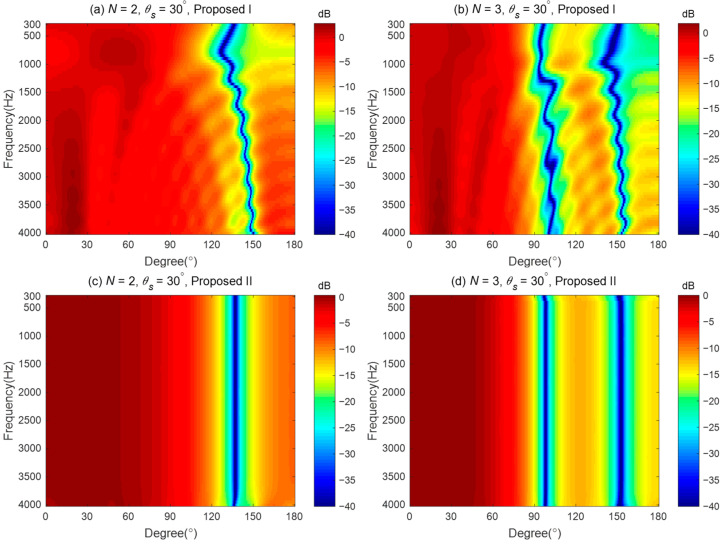
Different order target beampatterns synthesized by the proposed methods: (**a**) Second-order, Proposed I (**b**) Third-order, Proposed I (**c**) Second-order, Proposed II (**d**) Third-order, Proposed II.

The broadband synthesized radiation patterns are shown in Figure 4. Figure 4a,b show the broadband beampatterns of the second-order and third-order synthesized beampatterns of the proposed method I. There are two issues with the beampattern generated by the proposed method I. Firstly, the beampatterns cannot maintain consistency, as both the main lobe width and the null positions change with frequency. Secondly, the maximum values of the generated beampatterns deviate from the desired direction, which means that the generated beampattern does not closely approximate the desired beampattern. Figure 4c,d plot the broadband beampatterns synthesized using the proposed method II. As seen, the proposed method II can design the different orders of target radiation patterns. The resulting beampatterns almost maintain frequency-invariance across the evaluated frequency range.

**Figure 5:**
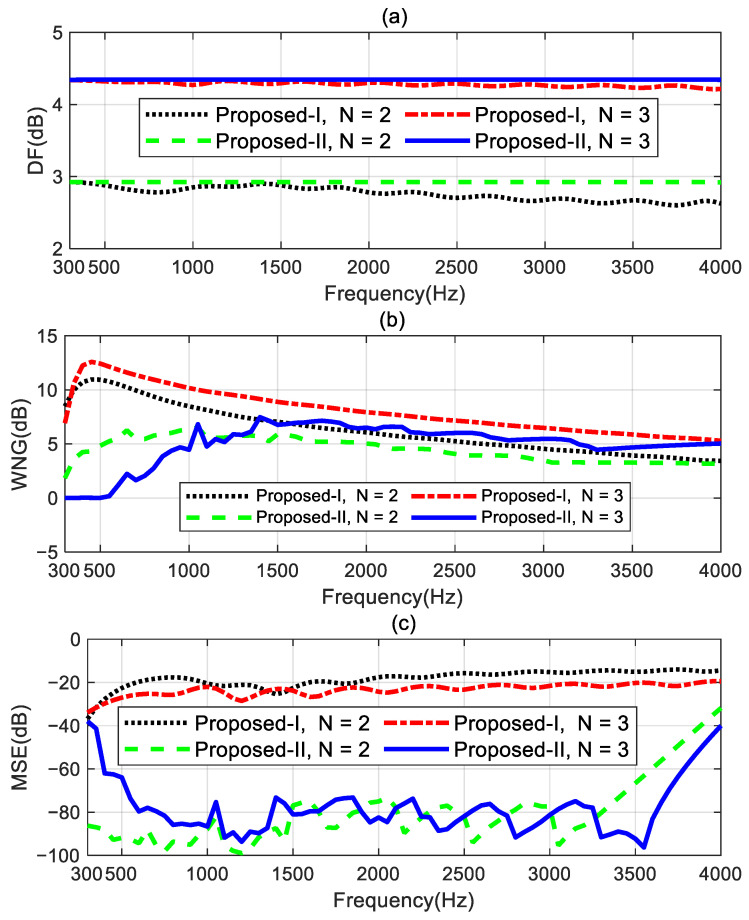
Performance comparison of the proposed methods for designing different order target beampatterns: (**a**) DF, (**b**) WNG, and (**c**) MSE.

The performance comparisons of the two proposed methods for designing the second- and third-order target differential beampatterns are shown in Figure 5. The performance measures are DF, WNG, and MSE. Figure 5a shows the DF increases while the order of the target beampattern increases. The DF of the proposed method I cannot remain at constant values and exhibits some fluctuations at certain frequencies. However, the proposed method II can maintain constant DF values over the frequency range of interest (300 Hz–4 kHz) for designing the different order target beampatterns. This indicates the proposed method II can achieve the frequency-invariant beampatterns in the whole evaluated frequency range. From Figure 5b, the WNGs of the proposed method II are smaller than those of the proposed method I. This is because the proposed method I uses all the remaining degrees of freedom of the array to optimize the WNG while satisfying the linear constraints. Therefore, the proposed method I has the highest WNG. The proposed method II, while meeting the linear constraints and ensuring the WNG exceeds $\varepsilon_{\mathrm{WNG}}$, uses the remaining degrees of freedom of the array to optimize the MSE. The preset value $\varepsilon_{\mathrm{WNG}}$ plays a role in a trade-off between the WNG and the MSE. As can be seen in Figure 5c, the MSE of the proposed method II is significantly lower than that of the proposed method I. Especially in the range of 1–3.5 kHz, compared to the proposed method I, the proposed method II achieves a reduction of over 40 dB in MSE by only slightly decreasing the WNG value. It is interesting to note that the performance of the proposed method I in designing the third-order target beampattern is superior to its performance in designing the second-order target beampattern. It has a larger DF factor, higher WNG, and lower MSE. This means that the beamformer of the third-order ideal beampattern has stronger directivity, higher robustness, and lower mean square error. Nevertheless, the MSEs of the proposed method I are still only around −20 dB, which does not meet the requirements for practical applications. However, the proposed method II can significantly improve the mean square error by sacrificing a slight degree of robustness, which is highly valuable in real applications. Therefore, we will only study the performance of the proposed method II in the following sections. Any mention of the proposed method hereafter refers to the beamformer obtained by solving the optimization problem (55).

### 4.2. Impact of the Parameter $\varepsilon_{\mathrm{WNG}}$

$\varepsilon_{\mathrm{WNG}}$ is a key parameter to trade off the robustness and the approximation error of the proposed method. This simulation studies the impact of the value of $\varepsilon_{\mathrm{WNG}}$ on the performance of the proposed method, with the third-order differential beampattern (see in Figure 3b) being the ideal radiation pattern. The level of $\varepsilon_{\mathrm{WNG}}$ is set to four different values: (1) $\varepsilon_{\mathrm{WNG}}=\varepsilon_{\mathrm{WNG}}^{\max}$ dB; (2) $\varepsilon_{\mathrm{WNG}}=(\varepsilon_{\mathrm{WNG}}^{\max}-2)$ dB; (3) $\varepsilon_{\mathrm{WNG}}=0$ dB; and (4) $\varepsilon_{\mathrm{WNG}}=-10$ dB.

**Figure 6:**
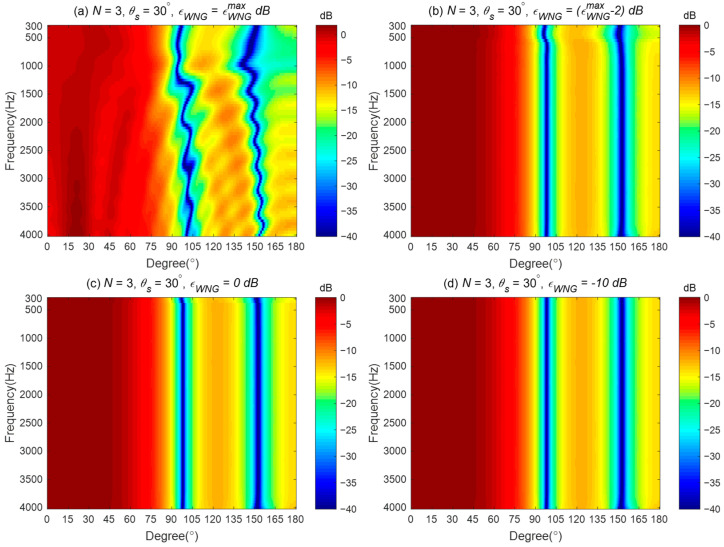
Broadband beampatterns of the third-order differential beampattern with a line array for different $\varepsilon_{\mathrm{WNG}}$ values: (**a**) $\varepsilon_{\mathrm{WNG}}=\varepsilon_{\mathrm{WNG}}^{\max}$ dB, (**b**) $\varepsilon_{\mathrm{WNG}}=(\varepsilon_{\mathrm{WNG}}^{\max}-2)$ dB, (**c**) $\varepsilon_{\mathrm{WNG}}=0$ dB, and (**d**) $\varepsilon_{\mathrm{WNG}}=-10$ dB.

The corresponding broadband radiation patterns of the proposed method are shown in Figure 6. Figure 6a presents the synthesized beampattern when $\varepsilon_{\mathrm{WNG}}$ equals $\varepsilon_{\mathrm{WNG}}^{\max}$ dB. It gives the same result shown in Figure 4b, indicating the solutions obtained by solving the optimized problem in (55) equal the solution in (46) when $\varepsilon_{\mathrm{WNG}}=\varepsilon_{\mathrm{WNG}}^{\max}$ dB. Figure 6b displays the synthesized beampattern when $\varepsilon_{\mathrm{WNG}}=(\varepsilon_{\mathrm{WNG}}^{\max}-2)$ dB. With a slight decrease in the value of $\varepsilon_{\mathrm{WNG}}$, the mean square error (MSE) between the synthesized beampattern and the ideal beampattern is reduced above 500 Hz (shown in Figure 6c), resulting in an improvement of the synthesized beampattern, although there is a slight deviation from the ideal beampattern at low frequencies. Furthermore, from Figure 6c,d, we can observe that as the value of $\varepsilon_{\mathrm{WNG}}$ is further reduced, the synthesized beampattern can be closer to the ideal one across the entire evaluated frequency range.

**Figure 7:**
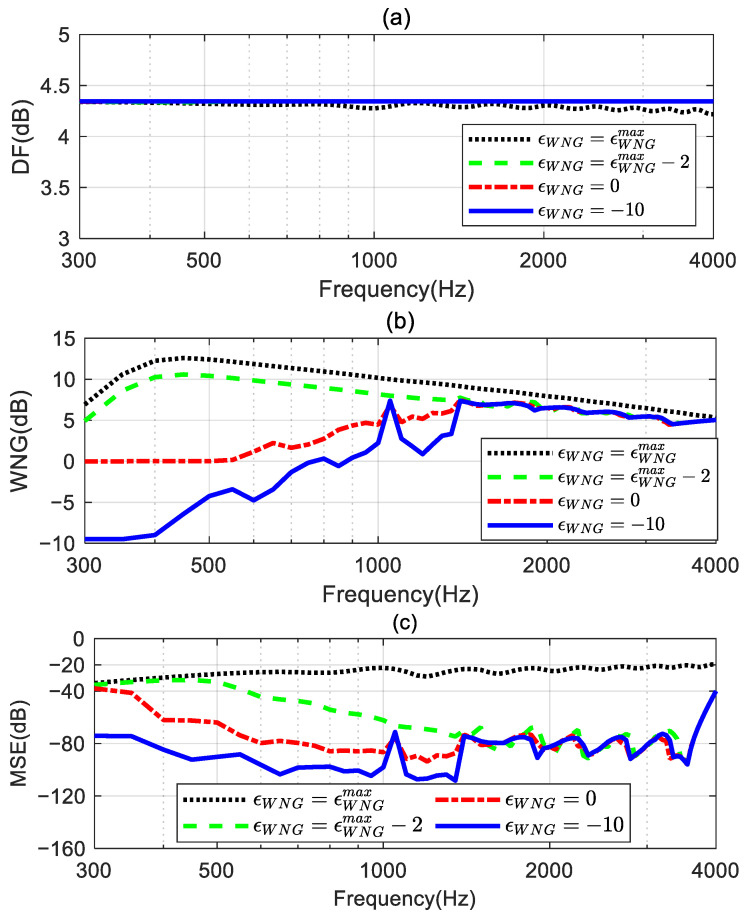
Performance comparison of the proposed methods with different $\varepsilon_{\mathrm{WNG}}$ values: (**a**) DF, (**b**) WNG, and (**c**) MSE.

The DFs, WNGs, and MSEs of these synthesized third-order beamformers are shown in Figure 7. Figure 7a shows that the DF of the beamformer with $\varepsilon_{\mathrm{WNG}}=\varepsilon_{\mathrm{WNG}}^{\max}$ fluctuates at certain frequencies, and the DFs of the beamformers with other $\varepsilon_{\mathrm{WNG}}$ values can maintain a constant over the whole evaluated frequencies range. This indicates that the frequency-invariant beampattern can be achieved by the proposed method with an appropriate $\varepsilon_{\mathrm{WNG}}$ value. Figure 7b,c plot the WNG and the MSE of the proposed method with different $\varepsilon_{\mathrm{WNG}}$ values. It can be observed that at low frequencies, especially below 1.4 kHz, the WNG and MSE of the beamformer decrease as $\varepsilon_{\mathrm{WNG}}$ decreases. This means that, with the decrease in the value of $\varepsilon_{\mathrm{WNG}}$, the synthesized beampattern more closely approaches the target beampattern, but this improvement comes at the expense of a decrease in the WNG. Above 1.4 kHz, the WNG of these beamformers does not differ significantly. However, the MSE of the beamformers when $\varepsilon_{\mathrm{WNG}}=\varepsilon_{\mathrm{WNG}}^{\max}-2$, $\varepsilon_{\mathrm{WNG}}=0$, and $\varepsilon_{\mathrm{WNG}}=-10$ is much smaller than the MSE of the beamformer when $\varepsilon_{\mathrm{WNG}}=\varepsilon_{\mathrm{WNG}}^{\max}$. Hence, we can tune the trade-off between the WNG and MSE of the beamformer by setting $\varepsilon_{\mathrm{WNG}}$ to slightly less than $\varepsilon_{\mathrm{WNG}}^{\max}$.

### 4.3. Impact of Loudspeaker Mismatch

It was assumed in the previous section that the loudspeakers used for designing the beamformer are ideal, without any mismatch issues among the loudspeaker units. However, in practice, there exists uncertainty in the loudspeaker characteristics (magnitude, phase, and position) due to the variations in the response of the drivers. This simulation investigates the impact of the driver mismatch on the proposed method with different $\varepsilon_{\mathrm{WNG}}$ values. The target beampattern and $\varepsilon_{\mathrm{WNG}}$ values are set the same as in Section 4.2. The perturbations are added to the spatial responses of the loudspeakers, for which the error has a multiplicative form with uniform distribution between −3 and +3 dB in magnitude and uniform distribution between −10° and +10° in phase. The performance measures are averaged over 1000 Monte Carlo trails.

**Figure 8:**
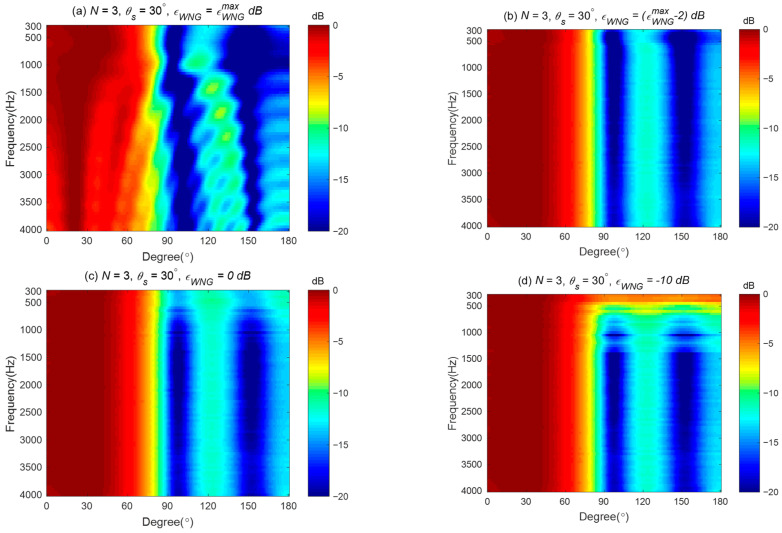
The averaged broadband beampatterns of the third-order differential beampattern with loudspeaker mismatch for different $\varepsilon_{\mathrm{WNG}}$ values: (**a**) $\varepsilon_{\mathrm{WNG}}=\varepsilon_{\mathrm{WNG}}^{\max}$ dB, (**b**) $\varepsilon_{\mathrm{WNG}}=\varepsilon_{\mathrm{WNG}}^{\max}-2$ dB, (**c**) $\varepsilon_{\mathrm{WNG}}=0$ dB, and (**d**) $\varepsilon_{\mathrm{WNG}}=-10$ dB.

Broadband beampatterns are plotted in Figure 8. By comparing Figure 8a,b with Figure 6a,b, we can observe that, aside from the difference in amplitude range, the basic shape of the beampattern remains unchanged. This indicates that when $\varepsilon_{\mathrm{WNG}}=\varepsilon_{\mathrm{WNG}}^{\max}$ and $\varepsilon_{\mathrm{WNG}}=\varepsilon_{\mathrm{WNG}}^{\max}-2$, the beamformer is robust enough to resist the perturbations in the frequency response of the loudspeaker. However, Figure 8c,d show significant differences from Figure 6c,d at low frequencies, suggesting that when $\varepsilon_{\mathrm{WNG}}=0$ and $\varepsilon_{\mathrm{WNG}}=-10$, the beamformer’s robustness is reduced, causing the synthesized beampattern to deviate from the target beampattern due to the errors added to the loudspeaker.

**Figure 9:**
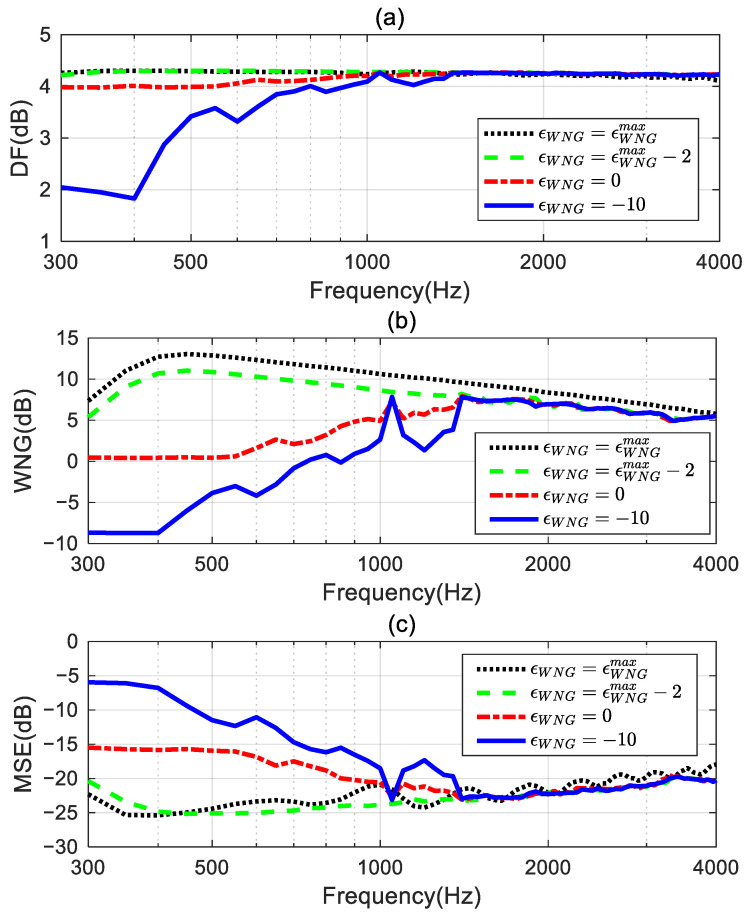
Performances of the proposed methods with different $\varepsilon_{\mathrm{WNG}}$ values considering the loudspeaker mismatch: (**a**) DF, (**b**) WNG, and (**c**) MSE.

Figure 9 shows the averaged DF, WNG, and MSE of the beamformers. Figure 9a shows that the DFs of the beamformers with $\varepsilon_{\mathrm{WNG}}=\varepsilon_{\mathrm{WNG}}^{\max}$ and $\varepsilon_{\mathrm{WNG}}=\varepsilon_{\mathrm{WNG}}^{\max}-2$ are almost identical over the whole evaluated frequency range. When $\varepsilon_{\mathrm{WNG}}=0$ and $\varepsilon_{\mathrm{WNG}}=-10$, the DFs decrease with the $\varepsilon_{\mathrm{WNG}}$ value decrease below 1 kHz. Figure 9b,c plot the WNG and MSE, respectively. Unlike the conclusion drawn in Section 4.2, that a smaller WNG of the beamformer results in a smaller MSE, when there exists a mismatch in the drivers, a smaller WNG value leads to a larger MSE, which means large deviations from the target beampattern at low frequencies. Hence, the appropriate selection of the $\varepsilon_{\mathrm{WNG}}$ value is crucial for the proposed method in practical applications.

### 4.4. Validation of the Steering Flexibility

In this simulation, we investigate the steering flexibility of the proposed method. For the broadside array illustrated in Figure 1, we consider four different desired directions: $\theta_{s}=30^{\circ }$, $\theta_{s}=45^{\circ }$, $\theta_{s}=60^{\circ }$, and $\theta_{s}=90^{\circ }$. The coefficients of the third-order differential beampattern with main lobe width equals 60°. The different desired directions need to be determined by solving (18) first, and then the synthesized beampattern is obtained using the proposed method. The parameter $\varepsilon_{\mathrm{WNG}}$ used in the simulation is set to $\left(\varepsilon_{\mathrm{WNG}}^{\max}-2\right)$ dB.

**Figure 10:**
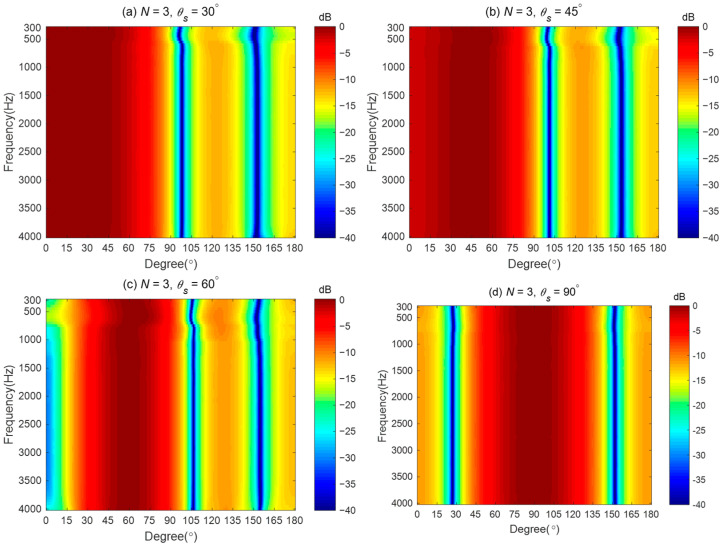
Broadband beampatterns of the third-order differential beampatterns for different steering directions: (**a**) $\theta_{s}=30^{\circ }$, (**b**) $\theta_{s}=45^{\circ }$, (**c**) $\theta_{s}=60^{\circ }$, and (**d**) $\theta_{s}=90^{\circ }$.

Figure 10 shows the synthesized broadband beampatterns. As seen, the main lobe of the synthesized beampatterns can be steered to the desired directions. This indicates that the proposed method can design different steerable beamformers with a linear array. The synthesized beampatterns slightly deviate from the ideal beampatterns at low frequencies for $\theta_{s}=30^{\circ }$, $\theta_{s}=45^{\circ }$, $\theta_{s}=60^{\circ }$, and $\theta_{s}=90^{\circ }$. When $\theta_{s}=90^{\circ }$, the proposed method can achieve a frequency-invariant beampattern over the whole evaluated frequency range.

**Figure 11:**
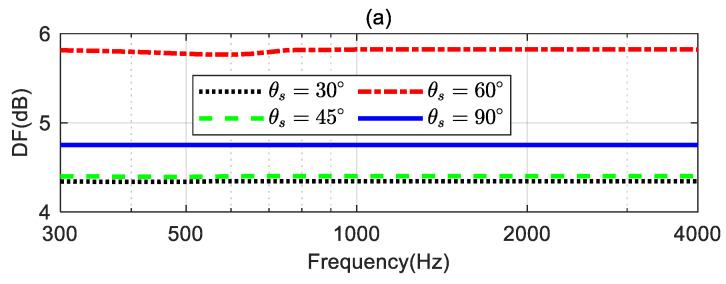

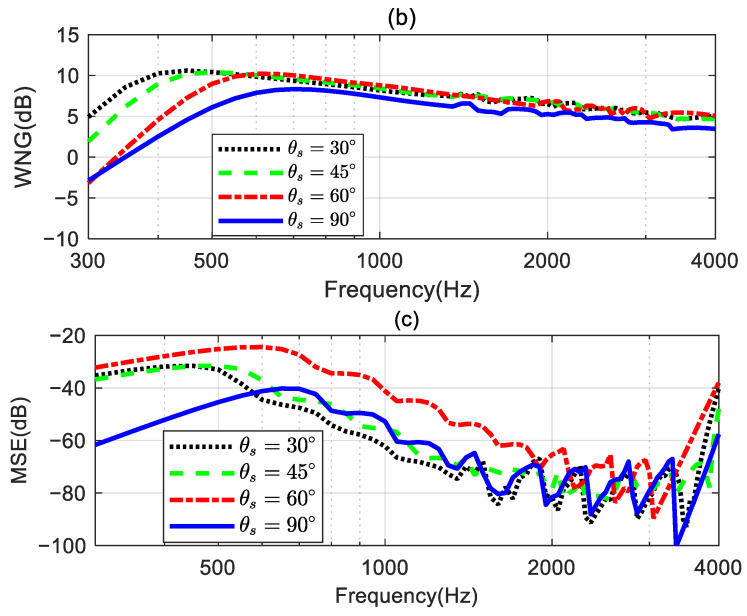
Performances of the proposed method with different desired directions: (**a**) DF, (**b**) WNG, and (**c**) MSE.

Figure 11 shows the DFs, WNGs, and MSEs of the beamformers with different desired directions. As seen from Figure 11a, when $\theta_{s}$ is 30°, 45°, and 60°, respectively, the DF of the beamformer increases as $\theta_{s}$ increases. However, when the desired direction $\theta_{s}$ is 90°, the DF does not increase as expected. That is because, in this case, the ideal beampattern is symmetrical with respect to the direction; the coefficient of the ideal beampattern satisfies $\alpha_{N,m}=0$ when m is odd, leading to a reduction in the DF value. From Figure 11b, the WNG decrease as $\theta_{s}$ increases at low frequencies. Figure 11c shows that when $\theta_{s}=90^{\circ }$, the MSE is below −40 dB over the whole evaluated frequency range, which is considered the ideal beampattern and has been well approximated.

### 4.5. Comparison with Other Steerable Beamforming Methods

To demonstrate the advantages of the proposed method in designing the steerable broadband differential beampattern with a linear array, we compare the performance of the proposed method with three other steerable beamforming methods: (i) the Delay and Sum (DS) method, which increases the acoustic energy in the desired direction by phase-aligning [45]; (ii) the Minimum Variance Distortionless Response (MVDR) method, which aims to minimize the output power of the array while maintaining a distortionless response in the desired direction [56]; and (iii) the Null Constrained (NC) method, which utilizes the null position for the ideal beampattern to design the differential beamformer [40]. The MVDR method requires a regularization parameter during the solving process. We set the regularization parameter to 0.001 in the simulation. The steerable differential beamformer we designed aims to approach the fourth-order differential beampattern with the main lobe width of 60° and the desired direction being steered to 120°.

**Figure 12:**
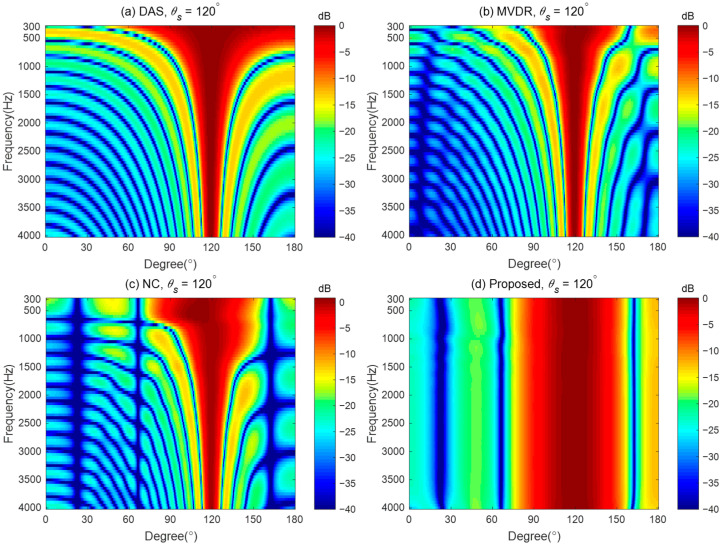
Broadband synthesized beampatterns of different beamforming methods: (**a**) the DS method, (**b**) the MVDR method, (**c**) the NC method, and (**d**) the proposed method.

Figure 12 plots the broadband beampatterns synthesized by these four methods. As seen, the main lobes of the DS and MVDR methods become narrower as the frequencies increase. The NC method can only maintain a constant main lobe width at low frequencies. Above 600 Hz, like the DS method, its main lobe width also narrows as the frequency increases. In contrast, the proposed method maintains the frequency-invariant beampattern over the whole frequency range.

**Figure 13:**
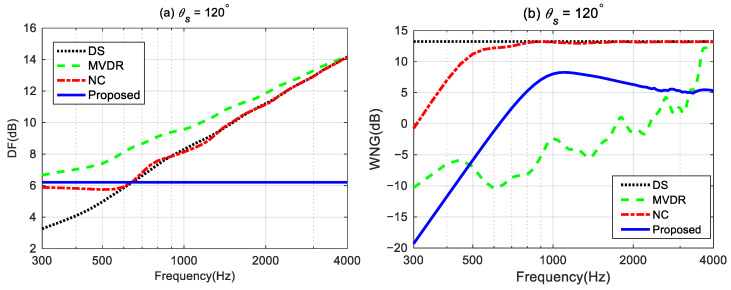
Performances of the different beamforming methods: (**a**) DF and (**b**) WNG.

The DFs and WNGs of the different beamforming methods are plotted in Figure 13. Figure 13a shows MVDR has the highest DF among the four methods, indicating the strongest ability of directivity across the whole frequency range. The DF of DS and MVDR increases with frequency. The DF of NC maintains almost the same below 600 Hz but increases with frequencies above 600 Hz, where the frequency-invariant cannot hold. Above 600 Hz, the DF of NC converges with that of the DS method. The DF of the proposed method can maintain a constant across the whole frequency range, indicating the frequency-invariant pattern can be achieved by the proposed method. It is worth noting that below 600 Hz, the DF of the DS method is the lowest, indicating that DS has the weakest directivity at low frequencies. In Figure 13b, DS has the maximum WNG and remains constant across the entire frequency range, which indicates that it has the strongest robustness for practical usage. The WNG of the NC method increases with frequency at low frequencies, and at high frequencies, its value matches that of the DS. In the 500–3k Hz frequency range, the MVDR method has the lowest WNG, indicating the worst anti-perturbation ability within this frequency band. Above 1 kHz, the WNG of the proposed method is lower than that of the DS and NC methods, but it is still greater than 5 dB, which is considered a proper level of robustness for practical usage. From the above discussion, it can be seen that, unlike the DS method, which has the highest WNG, and the MVDR method, which has the highest DF, the proposed method achieves a better balance between WNG and DF while generating frequency-invariant beampatterns.

## 5. Experiment and Discussion

**Figure 14:**
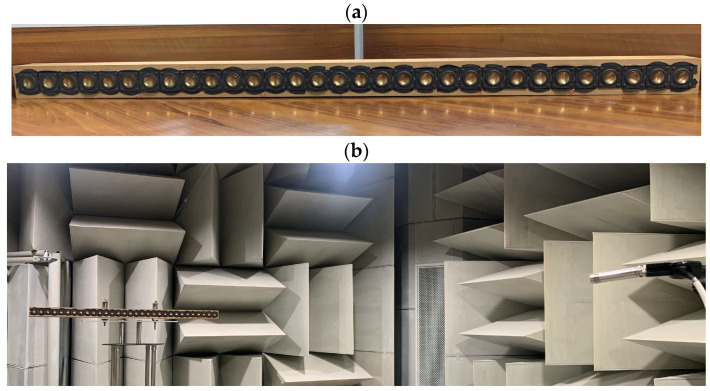
A photo of the linear array with 31 loudspeakers and experimental setup of the measurement: (**a**) the linear array and (**b**) the experimental setup.

Experiments were conducted in an anechoic chamber. A line loudspeaker array, shown in Figure 14a, was used. The array consists of 31 loudspeaker drivers, B1S (1 inch, metal cone, moving coil type) of HiVi Inc., spaced 3.8 cm apart. Each driver has an independent cavity with the internal dimensions of $3.6\times 5.8\times 6\;{\mathrm{cm}}^{3}$. The experimental setup is illustrated in Figure 14b. The array was mounted on a turntable and positioned in the front when the turntable angle was 0°. An omnidirectional microphone (BSWA MPA201, Beijing, China) was placed at 3 m from the geometrical center of the array. Both the microphone and the linear array were placed at a height of 2 m from the floor.

The transfer functions of the loudspeaker array have been measured to evaluate the performance of the different beamforming methods. The measured transfer functions include the acoustic characteristics, such as reflections and scattering effects due to the array cabinet, and loudspeaker mismatches, such as gain, phase, and position errors. Therefore, the beampattern using the measured transfer function is more aligned with practical applications than the free-field point source model, which does not take into account the influence of the array cabinet or the mismatch between loudspeaker drivers.

A linear swept sine signal in the frequency range of 300–4k Hz with a resolution of 5 Hz was reproduced by each loudspeaker to measure its transfer function. The signals recorded by the microphone were processed by an audio analyzer (Audio Precision 2720, Beaverton, OR, USA) to obtain the transfer function at a specific angle. The turntable was then rotated by 5° in the counter-clockwise direction, and the same measurement procedure was repeated. This process was repeated when the array was rotated from 0° to 180°, giving a total of 37 positions with 31 measurements each. Finally, the transfer functions were used to calculate the measured beampattern to evaluate the performance of the proposed method.

**Figure 15:**
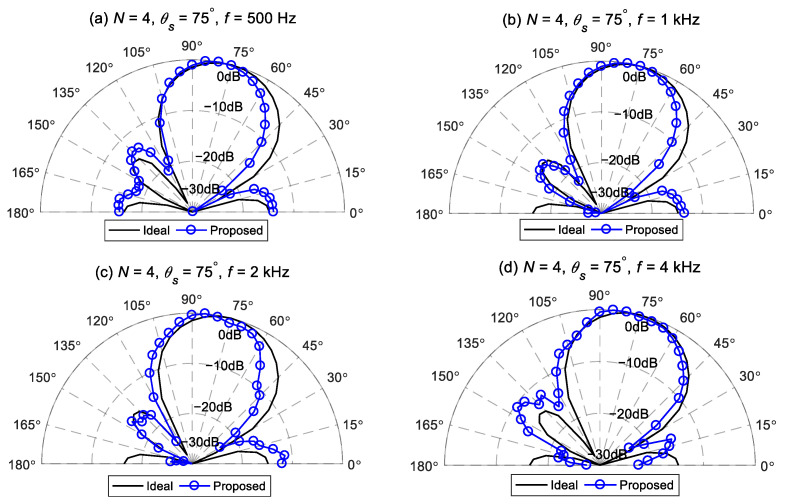
The measured beampattern of the proposed method and the ideal fourth-order differential beampattern at different frequencies for $\Delta =60^{{}^{\circ}},\theta_{s}=75^{{}^{\circ}}$: (**a**) 500 Hz, (**b**) 1 kHz, (**c**) 2 kHz, and (**d**) 4 kHz.

**Figure 16:**
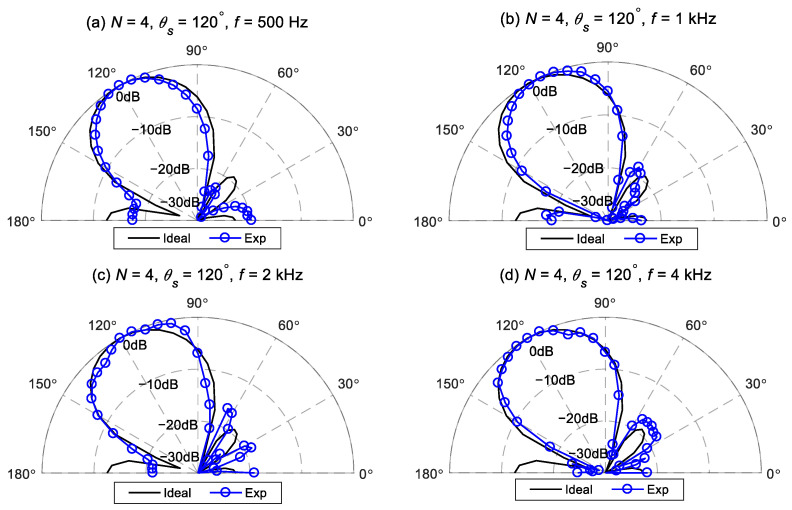
The measured beampattern of the proposed method and the ideal fourth-order differential beampattern at different frequencies for $\Delta =60^{{}^{\circ}},\theta_{s}=120^{{}^{\circ}}$: (**a**) 500 Hz, (**b**) 1 kHz, (**c**) 2 kHz, and (**d**) 4 kHz.

Figure 15 and Figure 16 illustrate comparisons between the measured beampatterns and the ideal beampatterns under different desired directions at frequencies: 500 Hz, 1 kHz, 2 kHz, and 4 kHz. The ideal beampattern has an order of 4, with a main lobe width of 60°. As can be seen, when the desired direction is at 75° or 120°, the measured beampatterns closely resemble the ideal beampattern, although there exist some minor differences due to array imperfections and measurement errors.

**Figure 17:**
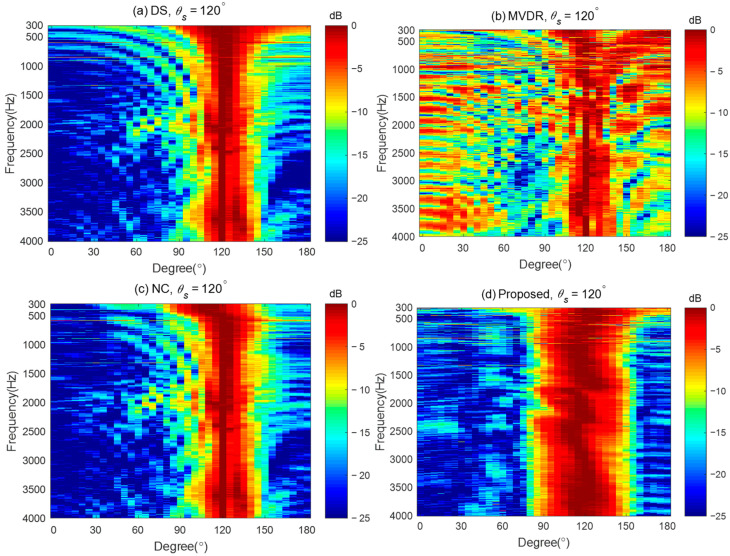
The measured broadband beampatterns of different beamforming methods: (**a**) the DS method, (**b**) the MVDR method, (**c**) the NC method, and (**d**) the proposed method.

To further demonstrate the good performance of the proposed method, Figure 17 gives the measured broadband beampatterns of the four different methods: DAS, MVDR, NC, and the proposed method. The regularization parameter used in the MVDR method is 0.001, and the WNG constraint value of the proposed method is set to $\varepsilon_{\mathrm{WNG}}^{\max}-2$ over the whole evaluated frequency range. It can be observed that the main lobe of the beampattern generated by the DS method becomes narrower as the frequency increases. The MVDR method cannot generate an effective beampattern due to its poor robustness. The main lobe of the NC method is well-maintained at low frequencies. However, at high frequencies, its beampattern becomes similar to that of the DS method, with the main lobe narrowing as the frequency increases. In contrast, the main lobe of the proposed method stayed almost the same in the evaluated frequency range of up to 4 kHz, which demonstrates that the frequency-invariant beampattern can be synthesized by the proposed method in a broadband of frequencies. It is worth noting that, compared to other existing beamforming methods, the advantage of the proposed method is its ability to form steerable frequency-invariant beampatterns over a wide frequency range. However, the above analysis and results were obtained under free-field conditions. The directivity of the proposed method is only validated in an anechoic chamber. In a regular room, room effect may degrade the directional performance of the proposed method. Future work will investigate the proposed method in more complex and real-world environments.

## 6. Conclusions

In this paper, we propose a method for designing a steerable frequency-invariant beamformer using a differential loudspeaker line array. The design process begins by determining the target differential beampatterns based on the desired direction, main lobe width, and beampattern order. These target beampatterns are then represented in the modal domain. The Jacobi-Anger series expansion is employed to design the beamformer, ensuring that the resulting beampattern closely aligns with the target differential beampattern. To further enhance beampattern matching and robustness, a multi-constraint optimization problem is formulated. This approach introduces a white noise gain constraint value, enabling a trade-off between the white noise gain performance and the mean square error of the synthesized beampatterns. We also derive the upper limit for the proposed constraint value. Simulation results demonstrate that setting the proposed constraint value slightly below this upper limit can significantly improve the mean square error performance. Both simulations and experimental results demonstrate that the proposed method outperforms existing steerable beamforming techniques, achieving steerable frequency-invariant beamforming across the frequency range of 300 Hz to 4 kHz.

## Data Availability

Data are available upon request.
